# Predictive Factors for Pancreatic Cancer and Its Early Detection Using Special Pancreatic Ultrasonography in High-Risk Individuals

**DOI:** 10.3390/cancers13030502

**Published:** 2021-01-28

**Authors:** Junko Fukuda, Kenji Ikezawa, Miho Nakao, Suetsumi Okagaki, Reiko Ashida, Tatsuya Ioka, Ryoji Takada, Takuo Yamai, Nobuyasu Fukutake, Hiroyuki Uehara, Shigenori Nagata, Hidenori Takahashi, Takahiro Tabuchi, Sachiko Tanaka, Kazuyoshi Ohkawa, Kazuhiro Katayama

**Affiliations:** 1 Department of Cancer Survey and Gastrointestinal Oncology, Osaka International Cancer Institute, Osaka 541-8567, Japan; fukuda-ju@mc.pref.osaka.jp (J.F.); nakao-mi@mc.pref.osaka.jp (M.N.); kanis@opho.jp (S.O.); rashida@goo.jp (R.A.); ioka-ta@umin.ac.jp (T.I.); sachi686@cocoa.plala.or.jp (S.T.); k.katayama@hosp.kaizuka.osaka.jp (K.K.); 2 Department of Hepatobiliary and Pancreatic Oncology, Osaka International Cancer Institute, Osaka 541-8567, Japan; ryoji.takada@oici.jp (R.T.); takuo.yamai@oici.jp (T.Y.); fukutake-no@mc.pref.osaka.jp (N.F.); uehara@oici.jp (H.U.); kazuyoshi.ohkawa@oici.jp (K.O.); 3 Department of Diagnostic Pathology and Cytology, Osaka International Cancer Institute, Osaka 541-8567, Japan; shnagata@oici.jp; 4 Department of Surgery, Osaka International Cancer Institute, Osaka 541-8567, Japan; takahasi-hi@mc.pref.osaka.jp; 5 Cancer Control Center, Osaka International Cancer Institute, Osaka 541-8567, Japan; tabuti-ta@mc.pref.osaka.jp

**Keywords:** special pancreatic US, MPD growth rate, early detection, pancreatic cancer, IPMN

## Abstract

**Simple Summary:**

The early detection of pancreatic cancer is required to improve prognosis. We aimed to analyze the predictive factors of neoplastic progression in patients at high risk for pancreatic cancer and examined the efficiency of surveillance using transabdominal special ultrasonography focusing on the pancreas (special pancreatic US). On long-term surveillance with special pancreatic US of 498 patients who had main pancreatic duct (MPD) dilatation (≥2.5 mm) and/or pancreatic cysts (≥5 mm), neoplastic progression developed in 11 patients (2.2%). Findings of both MPD dilatation and pancreatic cysts, MPD growth (≥0.2 mm/year) and cyst growth (≥2 mm/year) during surveillance were risk factors for neoplastic progression. Periodic surveillance using special pancreatic US allowed the early diagnosis of neoplastic progression (stage 0/I: 72.7%), leading to a favorable prognosis (overall survival: 8.8 years). This study clarified the efficiency of surveillance with special pancreatic US for high-risk individuals and key changes during surveillance to watch for.

**Abstract:**

Because pancreatic cancer has a dismal prognosis, a strategy for early diagnosis is required. This study aimed to identify predictive factors of neoplastic progression in patients at high risk for pancreatic cancer and examined the efficiency of surveillance using transabdominal special ultrasonography focusing on the pancreas (special pancreatic US). Patients with slight main pancreatic duct (MPD) dilatation (≥2.5 mm) and/or pancreatic cysts (≥5 mm) were enrolled in a prospective surveillance study with special pancreatic US in a Japanese cancer referral center. A total of 498 patients undergoing surveillance for ≥3 years were included. During the median follow-up of 5.9 years, neoplastic progression developed in 11 patients (2.2%), including 9 patients who underwent pancreatectomy. Eight patients (72.7%) were diagnosed with stage 0/I disease, with an overall survival duration of 8.8 years. Findings of both MPD dilatation and pancreatic cysts at initial surveillance, MPD growth (≥0.2 mm/year) and cyst growth (≥2 mm/year) during surveillance were identified as independent risk factors for neoplastic progression. In summary, surveillance with special pancreatic US for high-risk individuals contributed to earlier detection of neoplastic progression, leading to a favorable prognosis. During surveillance, attention should be paid to MPD growth as well as to cyst growth.

## 1. Introduction

The number of pancreatic cancer (PC) patients is increasing, and PC has become the third leading cause of cancer-related death in the United States [1,2]. PC has a poor prognosis, with a 5-year overall survival rate of 9% [1]. Approximately 80% of PC patients are diagnosed with metastatic or locally advanced disease because PC causes no or nonspecific symptoms in the early stage [3]. On the other hand, the 5-year survival rate of patients with tumors smaller than 10 mm (TS1a) was 80.4%, and that of patients with UICC stage 0 disease reached 85.8% [4,5]. Therefore, the development of a strategy for early diagnosis is required for improving the prognosis of PC [6].

There are several risk factors for PC, such as smoking history, chronic pancreatitis, diabetes mellitus and family history [7]. Based on a prospective follow-up, we previously reported that slight dilatation of the main pancreatic duct (MPD) (≥2.5 mm) and the presence of pancreatic cysts were also risk factors for the subsequent development of PC [8]. Therefore, periodic follow-up for patients who have slight MPD dilatation and/or pancreatic cysts could be a promising strategy for the early detection of PC.

Conventional transabdominal ultrasonography (US) is a useful screening examination for pancreatic diseases because of its minimal invasiveness. However, it has the following disadvantages. Its sensitivity for the detection of PC is approximately 75–89% [9], and its capability to detect PC is reduced by obesity or the presence of gastric gas [10]. To overcome these disadvantages of conventional US, we developed a special US examination focusing on the pancreas (hereafter referred to as special pancreatic US) [8,10,11,12,13,14]. Special pancreatic US is performed in the sitting position following the ingestion of liquid to reduce the effect of gastric gas [10].

Recently, Canto et al. reported that in a long-term follow-up study of high-risk individuals who had genetic factors of family history, most pancreatic ductal adenocarcinomas (PDACs) (9/10) were determined to be resectable [15]. They also identified radiologic features related to neoplastic progression. Although radiologic changes could be an important clue to the earlier detection of neoplastic progression, the characteristics of radiologic changes associated with neoplastic progression remain to be established. This study aimed to identify risk factors for predicting progression in terms of changes in imaging findings during surveillance with special pancreatic US as well as baseline characteristics at initial surveillance among patients at high risk of PC (patients with slight MPD dilatation and/or pancreatic cysts). We also examined the efficacy of periodic follow-up using special pancreatic US for detecting neoplastic progression in early stages.

## 2. Materials and Methods

### 2.1. Study Design and Patients

**Figure 1:**
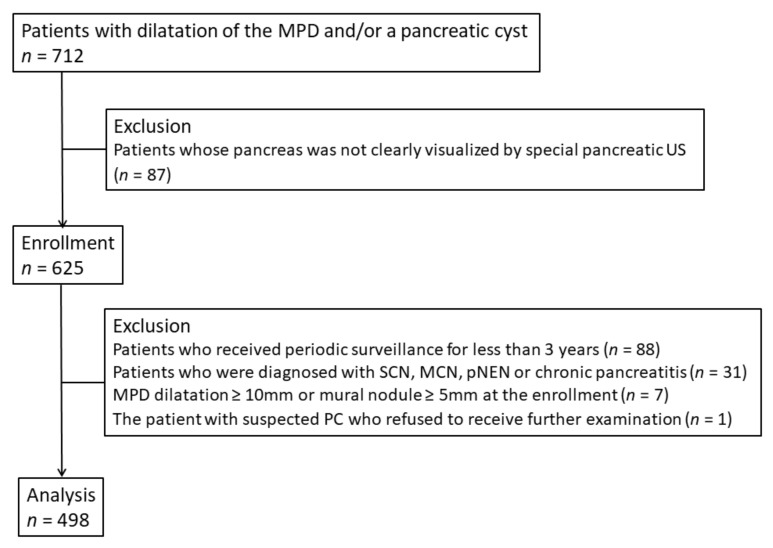
Flow chart of patient selection. MPD, main pancreatic duct; US, ultrasonography; SCN, serous cystic neoplasm; MCN, mucinous cystic neoplasm; pNEN, pancreatic neuroendocrine neoplasm; PC, pancreatic cancer.

From 1998, we performed a prospective surveillance study with special pancreatic US (every 3–6 months) in patients with pancreatic diseases including MPD dilatation and pancreatic cysts in a Japanese cancer referral center. From June 2007 to December 2010, after excluding 87 patients whose pancreas was not clearly visualized by special pancreatic US, 625 patients with MPD dilatation and/or pancreatic cysts were enrolled in a new prospective surveillance study at our institution (Figure 1). Special pancreatic US was performed twice per year, and magnetic resonance imaging (MRI) was performed once per year. Written informed consent was obtained from all participants. The inclusion criteria were as follows:

MPD dilatation (≥2.5 mm) and/or pancreatic cysts (≥5 mm);

Age less than 80 years old;

Eastern Cooperative Oncology Group performance status score of 0 or 1;

No active or recent malignancies of other organs; and

Clear visualization of at least three of the five parts of the pancreas (uncinate process, head, body, body–tail, and tail) on special pancreatic US.

Clinical findings and imaging characteristics were tracked over time from enrollment (or April 2005 in patients who had already undergone periodic surveillance with special pancreatic US) to the last follow-up by prospective recording up to February 2014. At initial surveillance, special pancreatic US, blood chemistry, and tumor marker tests were performed in all subjects.

Special pancreatic US was performed by following a previously published protocol [8,10,11]. With the patient in the sitting position (the Fowler position) for more than 20 min, at least 12 standard images of the pancreas were obtained before and after the ingestion of 350 mL of liquid to observe the whole pancreas according to a manual of procedures for decreasing inter-examiner differences. The Fowler position is the standard position during special pancreatic US because the liver descends to the pancreas and serves as an acoustic window for clear visualization of the pancreas. To allow patients to maintain this position without increasing abdominal muscle pressure or without feeling stress, we used an examination table with an adjustable backrest set at an approximately 60-degree angle. After screening the pancreas by changing the patient’s position, such as to the right lateral decubitus position and the left lateral decubitus position, the liquid-filled stomach method was used to eliminate the effect of gastric gas and to improve visualization of the pancreatic body–tail. At our institution, the patients ingested 350 mL of commercially available black tea with milk or green tea.

The MPD diameter was measured at the body of the pancreas on the magnified image before and after ingestion, and the larger size was used as the baseline value. The cyst size was defined as the major axis diameter. Special pancreatic US was conducted mainly by sonographers with ≥7 years of experience. The recorded images were reviewed by gastroenterologists with ≥5 years of experience. The major ultrasound diagnostic devices used for special pancreatic US were as follows: LOGIQ 7 (GE Healthcare, Milwaukee, WI, USA) with a 2–5.5 MHz wide-band convex probe; EUB-6500 and EUB-8500 (HITACHI, Tokyo, Japan); SSD5500 and SSD1700 (Aloka, Tokyo, Japan) with a 3–6 MHz wide-band convex probe; Aplio 500 and Aplio XG (TOSHIBA, Tokyo, Japan) with a 3–6 MHz wide-band microconvex probe.

Periodic examinations were primarily performed with only special pancreatic US, although MRI was also performed once per year in the new surveillance study from June 2007. When MRI was not indicated, contrast-enhanced computed tomography (CT) was alternatively used. According to each subject’s condition, the patients underwent detailed clinical examinations, including contrast-enhanced CT, MRI, endoscopic retrograde cholangiopancreatography (ERCP) with pancreatic juice cytology, endoscopic ultrasound (EUS), EUS-guided fine-needle aspiration (EUS-FNA), and/or other examinations. In the patients with neoplastic progression, the clinical course after diagnosis was followed up with medical records at our institution and/or outside medical records, when applicable, up to June 2020. In the patients who underwent upfront pancreatectomy, the date of the procedure was determined as the starting point for evaluating survival. In the patients who underwent pancreatectomy after neoadjuvant therapy or those who did not undergo surgery, the date of treatment initiation for PC (chemotherapy) was defined as the starting point for the analysis of survival.

The endpoint was neoplastic progression defined as the development of pathologically proven PDAC, intraductal papillary mucinous neoplasm (IPMN) with associated invasive carcinoma and/or intraductal papillary mucinous neoplasm high-grade dysplasia (IPMN-HGD). The final diagnoses of these neoplasms were determined by pancreatic juice cytology during ERCP, EUS-FNA or surgical pathology. Pathological diagnoses were confirmed by an expert pathologist (S.N.), and pretherapeutic staging was performed according to the UICC staging system (8th edition) [16].

Because this study evaluated the cumulative incidence of neoplastic progression and identified its predictors during surveillance, we excluded patients who satisfied the following conditions: (1) periodic surveillance for less than 3 years after the initiation of surveillance (*n* = 88); (2) a diagnosis of serous cystic neoplasm, mucinous cystic neoplasm, pancreatic neuroendocrine neoplasm or chronic pancreatitis on imaging and/or pathology (*n* = 31); (3) MPD dilatation ≥10 mm or a mural nodule (≥5 mm) detected at surveillance initiation (*n* = 7); and (4) the presence of suspected PC during surveillance with patient refusal to undergo further examination (*n* = 1). After excluding these patients from the 625 enrolled patients, the data of 498 remaining patients, including the patients with IPMN, were used for the present analysis (Figure 1). The present study was approved by the Institutional Review Board at Osaka International Cancer Institute (No. 1802069357-2) and performed in accordance with the Declaration of Helsinki.

### 2.2. Statistical Analysis

Categorical variables are described as percentages, and continuous variables are presented as the median and interquartile range (IQR). Patient characteristics were compared using Fisher’s exact test for categorical variables or the Mann–Whitney U-test for continuous variables. The MPD diameter, number of cysts and maximal cyst diameter were collected using special pancreatic US.

To examine factors associated with neoplastic progression, hazard ratios (HRs) and 95% confidence intervals (CIs) were calculated using the proportional hazards model for competing risks [17]. Factors with a *p*-value < 0.10 on univariate analysis were entered into the multivariate logistic regression models. The MPD growth rate (diameter difference from the initial to the last surveillance visit/follow-up period [mm/year]) and the cyst growth rate (size difference from the initial to the last surveillance visit/follow-up period [mm/year]) were calculated. Patient survival time was analyzed using the Kaplan–Meier method, and differences were evaluated using the log-rank test. Statistical analyses were performed using EZR (Saitama Medical Center, Jichi Medical University, Saitama, Japan), a graphical interface for the R Commander software package for Windows (version 1.50) [18]. *p* values less than 0.05 were considered statistically significant.

## 3. Results

### 3.1. Patient Characteristics

The characteristics of the 498 patients included in the present study are summarized in Table 1. Two hundred five patients (41.2%) were men, and the median age was 65 years (IQR, 59–70 years). The median diameter of the MPD was 2.35 mm (IQR, 1.7–3.2 mm). The proportion of patients whose MPD was less than 5 mm in diameter was 95.0% (473/498). The median number of pancreatic cysts was 1 (IQR, 1–2). The median maximal cyst diameter was 14.0 mm (IQR, 9.3–20.0 mm). The median levels of carbohydrate antigen 19-9 (CA19-9), pancreas amylase (P-amylase), elastase, alkaline phosphatase (ALP), and fasting blood sugar (FBS) were 12 IU/mL (IQR, 8–22 IU/mL), 31 IU/L (IQR, 25–38.75 IU/L), 112 ng/dL (IQR, 84–156 ng/dL), 213 IU/L (IQR, 177–262 IU/L) and 95 mg/dL (IQR, 88–101 mg/dL), respectively.

### 3.2. Cumulative Incidence of Neoplastic Progression

**Figure 2:**
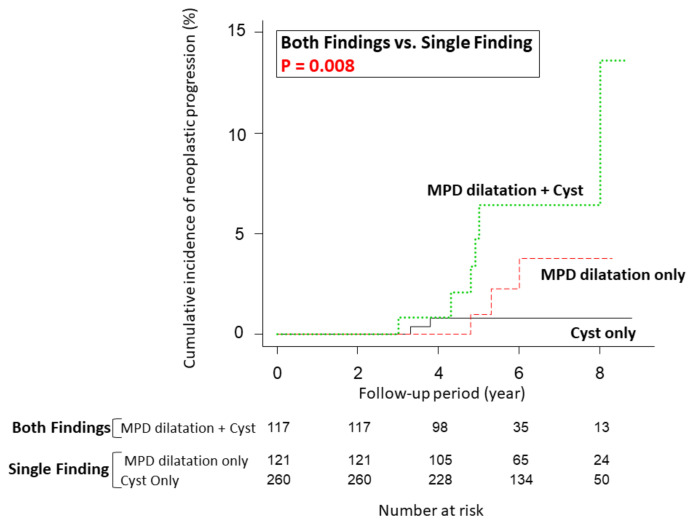
Comparison of the cumulative incidence of neoplastic progression in terms of MPD dilatation (≥2.5 mm) and the presence of pancreatic cysts (≥5 mm) at initial surveillance. The incidence at 5 years was 6.4% in patients with MPD dilatation (≥2.5 mm) and cysts, 1.0% in patients with only MPD dilatation, and 0.8% in patients with only pancreatic cysts. MPD, main pancreatic duct.

The median follow-up time for the entire cohort was 5.9 years (range: 3.0–8.8 years). Eleven of 498 patients (2.2%) had neoplastic progression. The cumulative incidence of neoplastic progression is shown in Supplementary Figure S1. The incidence at 5 years from initial surveillance was 2.0%, and the 8-year incidence was 3.9%. The median time from initial surveillance to neoplastic progression was 4.8 years (range: 3.0–8.0 years). The incidence in the patients with both findings of slight MPD dilatation (≥2.5 mm) and pancreatic cysts at initial surveillance was significantly higher than that in the patients with a single finding (either slight MPD dilatation or pancreatic cysts) (*p* = 0.008). At 5 years, the incidence in the patients with both findings was 6.4%, that in the patients with only MPD dilatation was 1.0%, and that in the patients with only pancreatic cysts was 0.8% (Figure 2).

**Figure 3:**
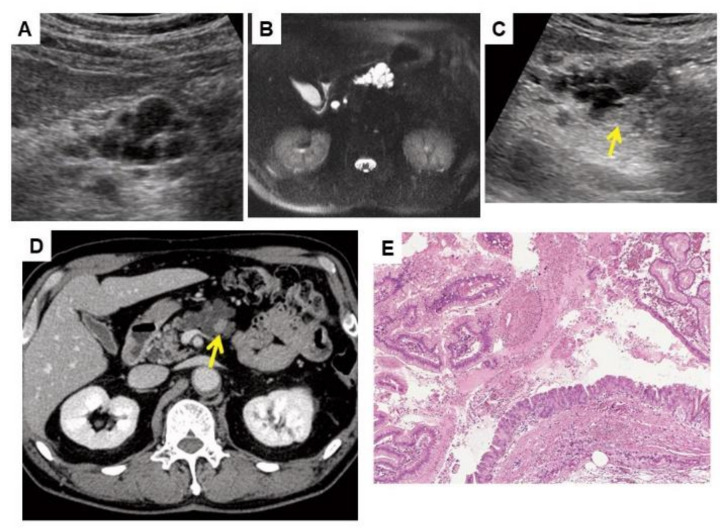
Images of a 62-year-old man who subsequently developed IPMN-HGD (patient No. 10 in Table 2). (**A**) Special pancreatic US at initial surveillance revealed a 40-mm multilocular cyst in the pancreatic body. (**B**) Abdominal MRI at initial surveillance revealed a multilocular cyst that was diagnosed as branched-duct IPMN. (**C**) Special pancreatic US 4.8 years after the initiation of surveillance revealed a 10 mm mural nodule (MN) (arrow) in the pancreatic cyst. The cyst size increased to 50 mm. (**D**) Contrast-enhanced CT revealed enhancement of the MN (arrow). (**E**) Histological examination revealed IPMN-HGD in the resected pancreas (total pancreatectomy; hematoxylin-eosin stain, original magnification ×100). The patient was still alive without recurrence 9.3 years after surgical resection.

Detailed information on these 11 patients with neoplastic progression is summarized in Table 2. The final diagnosis was PDAC in five patients (including one carcinoma-in-situ and two PDACs concomitant with IPMN), IPMN with associated invasive carcinoma in three patients and IPMN-HGD in three patients. Findings leading to a detailed examination were obtained by special pancreatic US in eight patients, MRI in one patient, contrast-enhanced CT in one patient and symptoms (weight loss and back pain) in one patient. Among the eight patients whose initial findings were obtained by special pancreatic US, the same findings were detected by CECT or MRI in three patients (No. 2, No. 3, and No. 10). On the other hand, in the other five patients, echoic lesions (masses or mural nodules) were not detected by CECT performed as a detailed examination (No. 4, No. 5, No. 6, No. 8, and No. 9). All cases of neoplastic progression were cytopathologically confirmed by pancreatic juice cytology during ERCP (*n* = 7) or EUS-FNA (*n* = 4). Among the 11 patients with neoplastic progression, two patients did not undergo surgery. One patient (No. 7) was diagnosed with resectable PDAC (cT2N0M0, stage IB), but liver metastasis was detected during neoadjuvant chemotherapy. The other patient (No. 1) underwent chemotherapy due to a diagnosis of locally-advanced unresectable cancer (IPMN with associated invasive carcinoma) (cT4N0M0, stage III). The remaining nine patients underwent pancreatectomy (upfront surgery, 7; surgery after neoadjuvant therapy, 2). These neoplasms were classified as stage 0 in four patients, stage IA in two patients, stage IB in one patient, stage IIB in one patient and stage III in one patient. In patient No. 10, cyst growth and the appearance of mural nodules were detected with special pancreatic US (Figure 3). After confirming malignancy with pancreatic juice cytology during ERCP, this patient underwent total pancreatectomy (final diagnosis: IPMN-HGD) and survived without recurrence. In summary, among 11 patients with neoplastic progression, 8 patients (72.7%) had UICC stage 0 or I disease.

**Figure 4:**
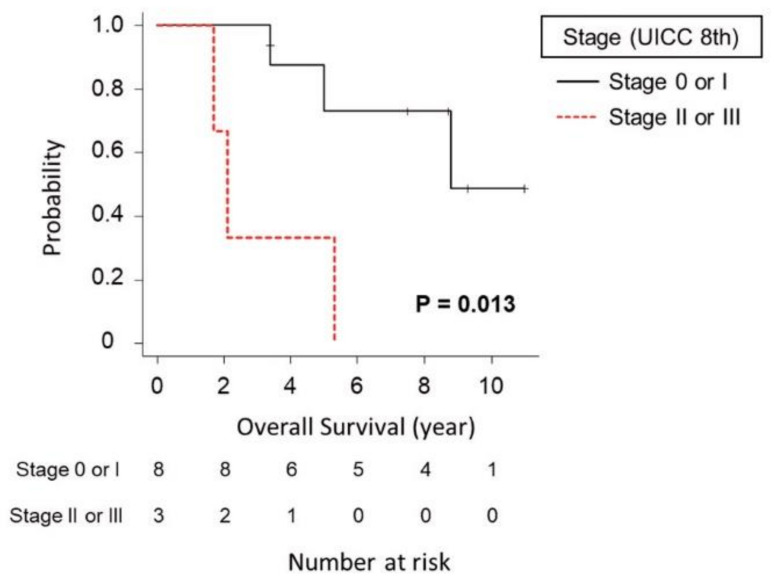
Comparison of overall survival between patients with neoplastic progression who were diagnosed with stage 0 or I disease and those who were diagnosed with stage II or III disease. The median overall survival time of the eight patients who were diagnosed with UICC stage 0 or I disease was significantly longer than that of the three patients who were diagnosed with UICC stage II or III disease (8.8 years vs. 2.1 years (*p* = 0.013)).

Among the patients with neoplastic progression, the median overall survival time was 8.8 years (Supplementary Figure S2). The median overall survival time of the eight patients who were diagnosed with UICC stage 0 or I disease was significantly longer than that of the three patients who were diagnosed with UICC stage II or III disease (8.8 years vs. 2.1 years (*p* = 0.013)) (Figure 4). There was no significant difference in overall survival between the six patients with neoplastic progression derived from IPMN and the other five patients with PDAC (including two PDACs concomitant with IPMN) (*p* = 0.805).

### 3.3. Factors Associated with Neoplastic Progression

Baseline patient characteristics at surveillance initiation were compared between progressors and non-progressors (Table 1). The MPD diameter was significantly larger in progressors than in non-progressors (median (IQR), 3.8 mm (2.7–9.2 mm) vs. 2.3 mm (1.7–6.3 mm); *p* = 0.005). The FBS level was significantly higher in progressors (median (IQR), 100 mg/dL (95–116 mg/dL) vs. 95 mg/dL (88–101 mg/dL); *p* = 0.030). There were no significant differences in age, sex, cyst number, maximum cyst diameter, or CA19-9, P-amylase, elastase or ALP levels. In terms of baseline characteristics, the univariate logistic regression analysis revealed that findings of both slight MPD dilatation and the presence of pancreatic cysts as well as the FBS level were significant risk factors for neoplastic progression (Table 3). On multivariate analysis, these two factors were identified as significant risk factors for neoplastic progression (Table 3). Findings of both slight MPD dilatation and pancreatic cysts at initial surveillance were significantly associated with neoplastic progression (HR: 5.095, 95% CI: 1.519–17.090, *p* = 0.008). A higher FBS level (≥95 mg/dL) was also revealed as a statistically significant factor associated with neoplastic progression (HR: 4.412, 95% CI: 1.027–18.950, *p* = 0.046).

On univariate analysis of factors associated with neoplastic progression in terms of changes in imaging findings during surveillance, the MPD growth rate (≥0.2 mm/year), an increase in the cyst number and the cyst growth rate (≥2 mm/year) were significant risk factors for neoplastic progression (Table 4). On multivariate analysis of these three factors, the MPD growth rate and the cyst growth rate were identified as independent risk factors for neoplastic progression (MPD growth rate, HR: 17.600, 95% CI: 3.547–87.310, *p* < 0.001; cyst growth rate, HR: 4.417, 95% CI: 1.240–15.740, *p* = 0.022) (Table 4).

## 4. Discussion

The purpose of the present study was to identify risk factors for predicting progression in patients with slight MPD dilatation (≥2.5 mm) and/or the presence of pancreatic cysts (≥5 mm) undergoing long-term surveillance with special pancreatic US. We demonstrated that findings of both MPD dilatation and the presence of pancreatic cysts at initial surveillance were identified as independent predictive factors of neoplastic progression. We previously showed that patients with findings of both slight MPD dilatation and pancreatic cysts had a higher risk of PC than patients with no finding [8]. While this previous study did not show a direct comparison of PC risk between the patients with both findings and those with a single finding (either slight MPD dilatation or the presence of pancreatic cysts), the present study demonstrates that findings of both slight MPD dilatation and pancreatic cysts constitute an independent predictive factor of neoplastic progression compared to a single finding. Although the risk of cumulative neoplastic progression in the current cohort was relatively low (2.2%), the 5-year cumulative rate among patients with both findings at initial surveillance was high (6.8%). Taken together with the results of our previous study [8], the present study demonstrates that patients with both findings are appropriate candidates for periodic follow-up with imaging modalities.

A high FBS level at initial surveillance was also identified as a significant factor for neoplastic progression. While diabetes mellitus is a well-known risk factor for PC, an increase in the FBS level has been suggested to be associated with an increased risk of PC [7,19,20,21]. The present study suggests that assessment of the FBS level before periodic surveillance could contribute to the prediction of pancreatic carcinogenesis.

Several guidelines on IPMN and pancreatic cysts have shown that the cyst growth rate is associated with an increased risk for high-grade dysplasia or PC; however, the cutoff for the cyst growth rate is still controversial because it varies among guidelines (≥5 mm/2 years in the Revised International Association of Pancreatology (IAP) Consensus guidelines; ≥5 mm/year in the guidelines of the European Study Group on Cystic Tumours of the Pancreas; and ≥3 mm/year in the guidelines of the American College of Gastroenterology (ACG)) [22,23,24,25]. In the current study, the cyst growth rate (≥2 mm/year) was identified as an independent risk factor for neoplastic progression, which is consistent with previous reports showing that the cyst growth rate (≥2 mm/year) is a predictive factor of malignancy in patients with branch-duct IPMN (Br-IPMN) [26,27]. Collectively, these results suggest that smaller changes in cyst size than those defined in several guidelines should be watched for.

Limited data are available regarding the association between the MPD growth rate during surveillance and neoplastic progression. Yoshioka et al. showed that the 10-year incidence of PC was significantly higher in patients with MPD diameter increases of ≥2 mm during the initial 5-year observation period among patients with pancreatic cystic lesions [18]. In the patients with Br-IPMN without worrisome features (WF) and high-risk stigmata (HRS), the MPD growth rate (≥0.2 mm/year) was shown to be an independent predictor of WF/HRS development [28]. While guidelines on IPMN and pancreatic cysts indicate that MPD dilatation (≥5 mm) is a risk factor for PC, they do not mention the MPD growth rate during the surveillance period [22,23,24,29]. In the present study, which primarily consisted of patients with MPD of less than 5 mm in diameter (95.0%), the MPD growth rate (≥0.2 mm/year) was identified as a strong predictive factor of neoplastic progression on multivariate analysis. Although guidelines on IPMN and pancreatic cysts do not describe the risk of MPD growth, careful attention to the increases in the MPD diameter is required during surveillance even in patients with an MPD diameter of less than 5 mm.

Notably, surveillance in the present study allowed the early detection of neoplastic progression, contributing to a favorable prognosis. Previous studies on surveillance for IPMN and pancreatic cysts have not shown that surveillance is sufficiently successful in terms of early detection. In a study on the surveillance of Br-IPMN patients, 55.6% of the patients who developed PC (5/9) were diagnosed at an unresectable stage (stage III or IV) [30]. Oyama et al. reported that in patients with Br-IPMN who developed PC during follow-up, 33.8% (23/68) were diagnosed at stage 0/I [31]. In contrast, of the 11 patients with neoplastic progression in the present study, nine (81.8%) underwent pancreatectomy. Eight patients (72.7%) were diagnosed with stage 0/I disease, leading to a favorable prognosis (median overall survival: 8.8 years). These results indicate that the surveillance method described in the present study is an efficient strategy for the early detection of PC, yielding a favorable prognosis in patients with neoplastic progression.

In patients with neoplastic progression, special pancreatic US primarily led to a further examination by detecting changes over time. Initial findings for further investigation were obtained by special pancreatic US in 8 of 11 patients with neoplastic progression (72.7%). According to the revised IAP 2017, European and ACG guidelines, lifelong surveillance (until patients are no longer eligible for surgery) is recommended or suggested for patients with pancreatic cysts [22,23,24]. Therefore, for long-term surveillance in the aging patient population, less invasive and more efficient imaging modalities are expected to be proposed. EUS and MRI are recommended as the main imaging modalities for the surveillance of pancreatic cysts [22,23,24,32]. Although EUS is undoubtedly an efficient modality for the visualization of pancreatic cysts as well as the whole pancreas [33], EUS is an invasive modality because of the need for sedation and the occurrence of complications, such as bleeding and perforation [34]. On the other hand, special pancreatic US is a less invasive imaging modality because it does not require sedation or cause complications. Furthermore, special pancreatic US ingeniously overcomes the weaknesses of conventional abdominal US to clearly visualize the pancreas [10]. Meanwhile, it is noteworthy that the remaining three cases of neoplastic progression (27.3%) were initially detected by other modalities or symptoms. Therefore, periodic MRI examinations (approximately once a year) in combination with special pancreatic US would be a preferred approach to decrease the risk of overlooking neoplastic progression.

In the present study, special pancreatic US was performed primarily by experienced sonographers. It is difficult for inexperienced sonographers to clearly visualize the pancreas. During surveillance with special pancreatic US, sonographers need to detect the changes of MPD, those of cyst diameter and the appearance of hypoechoic masses and mural nodules. Therefore, after sufficient experience with conventional abdominal US, training for performing special pancreatic US might be necessary for a period of several months to two years.

The present study has limitations. The current study was an observational study of prospectively collected data at a single referral center. Another limitation is associated with the inclusion criteria. Patients whose pancreas could not clearly be visualized by special pancreatic US were not enrolled in this study. However, only 12.2% (87/712) were excluded from the enrollment of our study, suggesting that the majority of the patients with MPD dilatation and/or pancreatic cysts can undergo surveillance with special pancreatic US. It is supposed that obesity and the presence of gastric gas are the reasons for unclear visualization of the pancreas. In our previous study, the ability of special pancreatic US to visualize the whole pancreas was reduced by obesity or the presence of gastric gas because subcutaneous/visceral fat and gastric gas attenuate ultrasound waves [14]. These patients need to undergo other follow-up examinations, such as EUS or MRI.

## 5. Conclusions

In conclusion, the patients who satisfied findings of both slight MPD dilatation (≥2.5 mm) and the presence of pancreatic cysts at initial surveillance had a significantly higher risk of neoplastic progression. During surveillance, attention should be paid to MPD growth as well as to cyst growth. Follow-up using special pancreatic US is a safe and efficient strategy for the early detection of neoplastic progression and an improvement in prognosis.

## Figures and Tables

**Table 1:** Baseline characteristics of the study patients at initial surveillance.

		Total	Progressor	Non-Progressor	*p*-Value
		(*n* = 498)	(*n* = 11)	(*n* = 487)	
Age, median (IQR), years		65 (59–70)	65 (59–70)	65 (59–70)	0.784
Sex	Male, n (%)	205 (41.2)	4 (36.4)	201 (41.3)	>0.999
	Female, n (%)	293 (58.8)	7 (63.6)	286 (58.7)	
MPD, median (IQR), mm		2.35 (1.7–3.2)	3.8 (2.7–9.2)	2.3 (1.7–6.3)	**0.005**
Cyst	Median number	1 (1–2)	2 (0.5–2)	1 (1–2)	0.243
	Max cyst size median (IQR), mm	14.0 (9.3–20.0)	17.5 (15.0–22.5)	13.0 (9.2–20.0)	0.087
CA19-9, median (IQR), IU/mL		12 (8–22)	18 (12.5–23.75)	12 (8–21)	0.060
P-amylase, median (IQR), IU/L		31 (25–38.75)	22.5 (6–24)	31 (25–39)	0.107
Elastase, median (IQR), ng/dL		112 (84–156)	108 (72–169.5)	113 (84–156)	0.900
ALP, median (IQR), IU/L		213 (177.25–262)	217 (167–248.5)	213 (178–252.5)	0.441
FBS, median (IQR), mg/dL		95 (88–101)	100 (95–116)	95 (88–101)	**0.030**

IQR, interquartile range; MPD, main pancreatic duct; CA19-9, carbohydrate antigen 19-9; P-amylase, pancreatic amylase; ALP, alkaline phosphatase; FBS, fasting blood sugar. Statistically significant at *p* < 0.05.

**Table 2:** Characteristics of patients with neoplastic progression detected during surveillance with special pancreatic ultrasound.

Patient Number	Age, Years/Sex	Initial US			Last US			Findings Leading to a Detailed Examination			Diagnostic Method	Final Diagnosis	Management	c/pTNM Classification	Stage (UICC 8th)	OS, Year	Cause of Death
		Cyst		MPD	Cyst		MPD	Imaging Modality	Findings/Location	Time to Detection, Year							
		Number	Size		Number	Size											
1	79/M	2	18.0	5.1	5	20.0	9.8	CECT	Hypovascular tumor/head	4.9	ERCP/PJC	IPMN with associated invasive carcinoma	No surgery (Chemotherapy)	cT4N0M0	cStage III	Died, 2.1	Cancer progression
2	80/M	4	33.0	9.2	4	54.0	18.4	US	Hypoechoic mass/head	8.0	EUS-FNA	IPMN with associated invasive carcinoma	PD	pT3N2M0	pStage III	Died, 1.7	Cancer progression
3	75/F	0	-	4.3	0	-	7.5	US	Rapid MPD dilatation/body	6.0	ERCP/PJC	PDAC (in situ)	PD	pTisN0M0	pStage 0	Alive, 8.7	NA
4	74/F	2	16.0	3.4	4	35.0	3.5	US	Cyst growth with appearance of a mural nodule/head	3.0	ERCP/PJC	IPMN-HGD	PD	pTisN0M0	pStage 0	Died, 8.8	Other disease
5	71/F	2	12.0	2.2	5	19.0	3.6	US	Hypoechoic mass/head	3.8	EUS-FNA	IPMN with associated invasive carcinoma	PD	pT2N1M0	pStage IIB	Died, 5.3	Cancer progression
6	68/F	1	17.0	1.2	2	19.0	1.6	US	Hypoechoic mass/body	3.3	ERCP/PJC	IPMN-HGD	DP	pTisN0M0	pStage 0	Alive, 11.0	NA
7	70/F	3	19.0	4.4	4	27.0	2.7	Symptoms *	-	5.0	ERCP/PJC	PDAC concomitant with IPMN	No surgery (Chemotherapy)	cT2N0M0	cStage IB	Died, 3.4	Cancer progression
8	64/F	0	-	2.8	1	17.0	4.0	US	Hypoechoic mass/body-tail	5.3	EUS-FNA	PDAC	PD after NACRT	ycT1bN0M0 (ypT0N0M0 <no residual tumor>)	ycStage IA	Alive, 7.5	NA
9	64/F	0	-	2.6	0	-	5.1	US	Hypoechoic mass/body	4.8	ERCP/PJC	PDAC	PD	pT1bN0M0	pStage IA	Died, 5.0	Cancer progression
10	62/M	1	40.0	6.5	2	50.0	13.0	US	Cyst growth with appearance of a mural nodule/body	4.8	ERCP/PJC	IPMN-HGD	TP	pTisN0M0	pStage 0	Alive, 9.3	NA
11	61/M	2	11.0	3.8	3	27.0	5.7	MRI	Solid mass/tail	4.3	EUS-FNA	PDAC concomitant with IPMN	DP after NACRT	ycT2N0M0 (ypT1bN0M0)	ycStage IB	Alive, 3.4	NA

US, ultrasonography; MPD, main pancreatic duct; OS, overall survival; M, male; F, female; CECT, contrast-enhanced computed tomography; MRI, magnetic resonance imaging; ERCP, endoscopic retrograde cholangiopancreatography; PJC, pancreatic juice cytology; EUS-FNA, endoscopic ultrasound-guided fine-needle aspiration; IPMN, intraductal papillary mucinous neoplasm; HGD, high-grade dysplasia; PD, pancreatoduodenectomy; DP, distal pancreatectomy; NACRT, neoadjuvant chemoradiation; TP, total pancreatectomy; NA, not applicable. * Symptoms: weight loss and back pain.

**Table 3:** Univariate and multivariate analyses of factors associated with neoplastic progression in terms of baseline characteristics at initial surveillance.

Factor		Univariate		Multivariate	
		HR (95% CI)	*p* Value	HR (95% CI)	*p* Value
Age	<65 years	1			
	≥65 years	1.725 (0.502–5.935)	0.39		
Sex	Female	1			
	Male	0.850 (0.250–2.885)	0.79		
MPD dilatation (≥2.5 mm) and/or pancreatic cysts (≥5 mm)	Single finding (either MPD dilatation or cysts)	1		1	
	Both findings	5.082 (1.537–16.810)	**0.008**	5.095 (1.519–17.090)	**0.008**
Max cyst size	<15 mm	1			
	≥15 mm	3.742 (0.745–18.810)	0.11		
CA19-9	<15 IU/mL	1			
	≥15 IU/mL	2.162 (0.610–7.661)	0.23		
P-amylase	<30 IU/L	1			
	≥30 IU/L	0.469 (0.148–1.490)	0.2		
Elastase	<110 IU/mL	1			
	≥110 IU/mL	0.726 (0.226–2.337)	0.59		
ALP	<215 IU/mL	1			
	≥215 IU/mL	1.204 (0.363–3.988)	0.76		
FBS	<95 mg/dL	1		1	
	≥95 mg/dL	4.397 (0.998–19.37)	0.05	4.412 (1.027–18.950)	**0.046**

HR, hazard ratio; CI, confidence interval; MPD, main pancreatic duct; P-amylase, pancreatic amylase; ALP, alkaline phosphatase; FBS, fasting blood sugar. Statistically significant at *p* < 0.05.

**Table 4:** Univariate and multivariate analyses of factors associated with changes in imaging findings during surveillance in relation to neoplastic progression.

Factor		Univariate		Multivariate	
		HR (95% CI)	*p* Value	HR (95% CI)	*p* Value
MPD growth rate/year	<0.2 mm	1		1	
	≥0.2 mm	30.150 (7.055–128.800)	**<0.001**	17.600 (3.547–87.310)	**<0.001**
Increase in cyst number	No	1		1	
	Yes	4.793 (1.251–18.360)	**0.022**	3.339 (0.986–11.310)	0.053
Cyst size growth rate/year	<2 mm	1		1	
	≥2 mm	11.740 (3.342–41.260)	**<0.001**	4.417 (1.240–15.740)	**0.022**

HR, hazard ratio; CI, confidence interval; MPD, main pancreatic duct. Statistically significant at *p* < 0.05.

## Data Availability

The data presented in this study are available on request from the corresponding author. The data are not publicly available due to privacy issues.

## Supplementary Materials

The following are available online at https://www.mdpi.com/2072-6694/13/3/502/s1, Figure S1: The cumulative incidence of neoplastic progression among 498 patients in the study population. Figure S2: Overall survival time of 11 patients with neoplastic progression.

## Abbreviations

ACG	American College of Gastroenterology
ALP	alkaline phosphatase
Br	branch-duct
CA19-9	carbohydrate antigen 19-9
CI	confidence interval
CT	computed tomography
ERCP	endoscopic retrograde cholangiopancreatography
EUS	endoscopic ultrasound
FBS	fasting blood sugar
FNA	fine-needle aspiration
HGD	high-grade dysplasia
HR	hazard ratio
IAP	International Association of Pancreatology
IPMN	intraductal papillary mucinous neoplasm
IQR	interquartile range
MPD	main pancreatic duct
MRI	magnetic resonance imaging
PC	pancreatic cancer
PDAC	pancreatic ductal adenocarcinoma
US	ultrasonography
