# Supplementary material for: Predictive Factors for Pancreatic Cancer and Its Early Detection Using Special Pancreatic Ultrasonography in High-Risk Individuals
Source: Cancers (Basel). 2021 Jan 28;13(3):502. doi: 10.3390/cancers13030502 (PMC7865866; doi:10.3390/cancers13030502)
Supplement: Supplementary file 1 [file cancers-13-00502-s001.pdf]

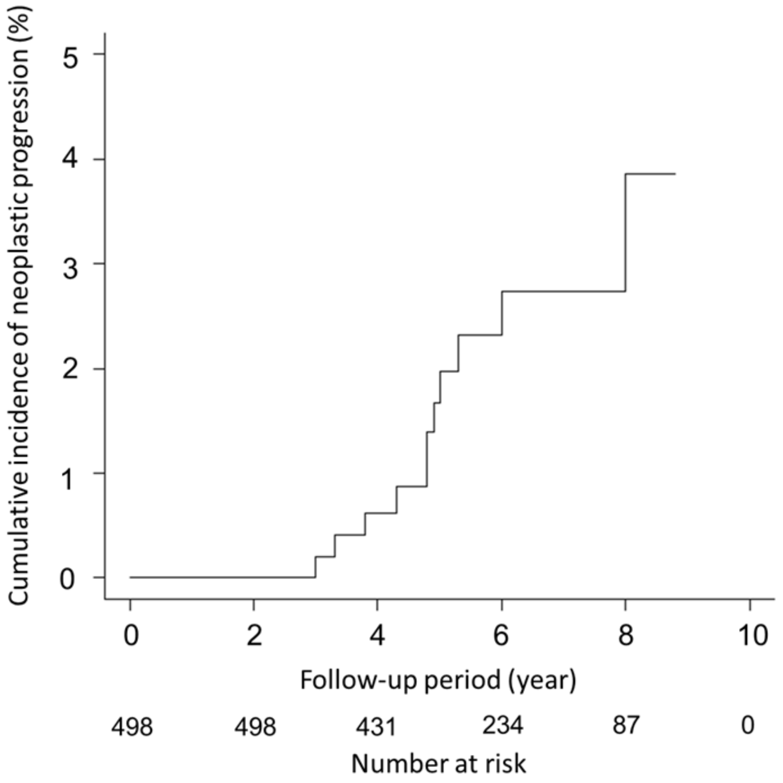

Figure 1. Cumulative incidence of neoplastic progression among 498 patients in the study population. The incidence at 5 years from initial surveillance was 2.0%, and the incidence at 8 years was 3.9%.

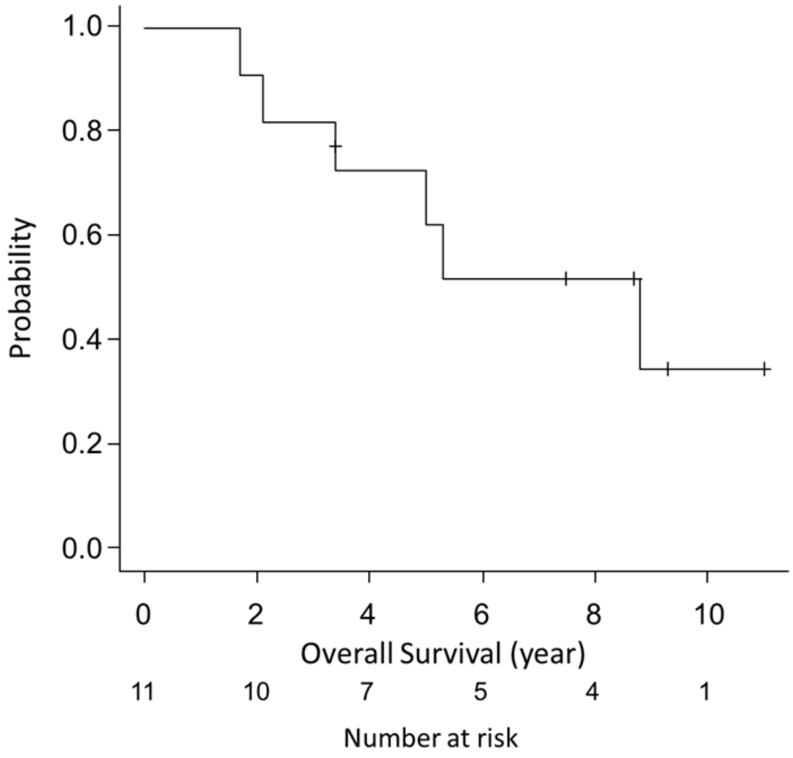

Figure S2. Overall survival time of eleven patients with neoplastic progression. The median overall survival time was 8.8 years
